# A thematic analysis of newly qualified doctors’ experiences of burnout

**DOI:** 10.1186/s12909-025-07076-z

**Published:** 2025-04-07

**Authors:** Colin R. Kilday

**Affiliations:** 1 Hull York Medical School, University of Hull, Hull, UK; 2 NHS Humber Health Partnership, Hull, UK

**Keywords:** Physician burnout, Postgraduate training, Burnout causes

## Abstract

**Background:**

Newly qualified doctors who are at the beginning of their professional careers are now at a significant risk of burnout. This is in-keeping with doctors in the UK who are concerned about developing burnout during the UK Foundation Programme (UKFP). The UKFP is the first role doctors have working within the National Health Service (NHS). This research aims to explore factors within the UKFP which contribute to newly qualified doctors experiences of burnout.

**Methods:**

Semi-structured interviews were conducted with 7 foundation doctors within one NHS trust to explore their experiences of burnout. A thematic analysis was then carried out using the data collected from these interviews.

**Results:**

The majority of doctors interviewed were able to discuss their own experiences of burnout and how it has impacted their role as a newly qualified doctor. Some doctors believe that working as a foundation doctor directly contributed to their experiences of burnout. Whereas other doctors considered wider issues within the NHS such as bullying and staffing issues to be contributing factors. In contrast, the community aspect of the UKFP was considered to be a protective factor against burnout.

**Conclusions:**

Burnout is a real concern amongst foundation doctors and many doctors will experience burnout at the beginning of their career. Therefore, further research is needed to identify effective support measures which can be implemented within the UKFP and used to support doctors as they begin their career in the NHS.

**Supplementary Information:**

The online version contains supplementary material available at 10.1186/s12909-025-07076-z.

## Background

Upon graduation from medical school, doctors begin a particularly stressful period of their lives when they take up their first role as a newly qualified doctor [1]. Within the United Kingdom this first role is incorporated into the UK Foundation Programme (UKFP). The UKFP is a 2-year programme where doctors continue their medical training after completing their medical degree [2]. Whilst these doctors are continuing their medical education within the programme, they are also taking up what is likely their first professional role working within the National Health Service (NHS). This can be challenging for foundation doctors as they try to manage their competing priorities; furthering their professional development, whilst simultaneously carrying out the day-to-day role of working as a doctor [3]. Many doctors feel that they are unprepared to take up this new role [4–5] As a result, there are concerns that this increases the risk of new doctors developing burnout at the very beginning of their careers [6].

Burnout is defined by the World Health Organization (WHO) as a syndrome caused by unmanaged chronic workplace stress. It is considered to have three core components which include, feelings of exhaustion, cynicism towards the job role and reduced professional efficacy [7]. Burnout within the medical profession is at an all-time high [8–9]. The General Medical Council (GMC) National Training Survey 2023 has identified that two thirds of doctors in training are now at a moderate to high risk of developing burnout. Doctors at an early stage in their careers in particular, are considered to be at a high risk of burnout [9].

This is perhaps unsurprising given previous research has highlighted that the transition from medical student to physician can often leave doctors feeling as if they have been “thrown in at the deep end.” [10–11]. Often this is attributed to the increasing workload doctors are now facing upon entering the workforce [12]. This has become particularly apparent within the NHS, as one in four junior doctors state that the demands of work are having a serious impact on their mental health and one third are currently experiencing a high level of burnout whilst at work [13–15]. Previous research has also recognised that newly qualified doctors have often yet to develop the resilience needed to ‘survive’ working in such a demanding environment [16–17]. Either these newly qualified doctors develop the coping mechanisms to carry out the role whilst under a great deal of stress or they burnout. Essentially they ‘sink or swim’ [12]. However, as the work demands increase, more pressure is being placed upon these newly qualified doctors, thus resulting in more doctors burning out [18].

Within the NHS, these high work demands are often now coupled with a lack of support from senior staff [19]. As newly qualified doctors enter the workforce they will often turn to seniors for guidance and teaching [20]. However, within the NHS, staff shortages remain a problem and senior doctors often find themselves overworked, leaving little time for mentoring junior doctors [21]. This can leave junior doctors feeling unsupported and isolated within the NHS workforce. As a result, they may feel they are making clinical decisions beyond their capabilities thus providing poor patient care [22]. The inability to provide appropriate patient care and the lack of available support are both recognised as contributing factors in physician burnout [18–19].

Upon the transition from medical student to physician, newly qualified doctors find themselves working within teams of doctors within the NHS. These teams have a hierarchical structure with newly qualified doctors occupying the lowest rung of the ladder. This leaves them vulnerable to bullying. Unfortunately, bullying remains a significant issue for doctors. Riley et al. (2021) found that over half of the foundation doctors they had interviewed had been bullied and experienced abuse [18]. This bullying was often instigated by consultants which often led to a tolerance of bullying throughout the clinical team [18, 23]. Bullying is widely accepted as a cause of burnout as it results in a persistent workplace stress placed upon the junior doctors which is often left unresolved [24–25].

More recently, doctors on the UKFP have also had to deal with additional issues whilst working within the NHS. Since the pandemic there has been significant changes made to the services that the NHS offers and it is clear that this has impacted foundation doctors training experience. Griffen et al. (2021) had noted that these service changes have resulted in changes to doctors rotas and increased the frequency in which doctors have to rotate round to different departments. This has led to increased levels of anxiety, loneliness and in some cases burnout of doctors in training [26]. Junior doctors in the UK also went on strike repeatedly throughout 2023–2024 period. The British Medical Association (BMA) state that the reasons why junior doctors went on strike were “A crippling cost-of-living crisis, burnout and well below inflation pay rises” [27]. Doctors who had chosen to strike were put under additional pressure and stress. This was because media outlets and politicians often stated that those choosing to strike would be harming patients and continued to call for doctors to return to work [28–29].

Whilst burnout has significant consequences for the physicians experiencing it, it also results in patients receiving poorer care. Research has found that doctors are more likely to make medical errors and patients are more likely to receive inadequate care if they are treated by doctors who are experiencing burnout [30–31]. According to the WHO, burnout is not considered a medical condition but is instead considered an “occupational phenomenon” which influences a worker’s health [7]. This is important as it places an emphasis on the employer, in this context the NHS, to identify those at risk and offer support to those experiencing burnout [32].

Whilst burnout remains a significant issue within the NHS, there may also be factors which are specific to the UKFP which cause burnout. ‘The F3 Phenomenon Report,’ already acknowledges that burnout is a real concern amongst doctors who have recently completed the UKFP. This report also acknowledges that post-foundation doctors have tried to reduce their own risk of burnout through taking a break from training. It also theorises that one potential way in which newly qualified doctors are attempting to reduce their risk of burnout is to cut their hours to less than full-time. The report states that this may “prevent or reduce burnout experienced through foundation training” [33].

As the risk of burnout grows greater amongst newly qualified doctors beginning work in an increasingly strained NHS, it is now more important than ever to understand this phenomenon. Foundation doctors are at the beginning their postgraduate careers and after only a relatively short period of time, many are experiencing burnout [9, 33]. Within the UK, this transition to newly qualified doctor takes place during the UKFP. However, there is currently limited research into the specific components of the UKFP which may be contributing to this occupational phenomenon and if there are any aspects of the UKFP which may be considered protective against burnout.

## Method

### Aim

This study sought to explore the training experiences of foundation doctors and factors within the UKFP which may have contributed to burnout.

### Study design

This study focussed on using a thematic analysis to explore foundation doctors lived experiences on the UKFP and how participants related their experiences on this programme to burnout. A thematic analysis was chosen as it can be used as a standalone method whilst it can also be used alongside a phenomenological methodology [34–35]. The purpose of phenomenological research is to understand an individual’s lived experiences within their own environment [36]. In this study, this meant attempting to understand the experiences of foundation doctors on the UKFP and how these experiences then related to foundation doctors own understanding and experiences of burnout.

As outlined previously, burnout often has a rigid definition or is classified based upon scoring systems such as the Maslach Burnout Inventory or Copenhagen Burnout Inventory [7, 37–38]. However, as burnout is increasingly being recognised within healthcare workers, foundation doctors are likely to have their own views and understanding of the term and what it means to them [12, 19, 38]. Therefore, it is important to examine how foundation doctors define and experience burnout in order to assess how it has been impacted by their role as a newly qualified doctor on the UKFP.

It should also be noted that this research took place during a time when there was growing discontent amongst doctors in training. This was due to ongoing junior doctor strikes and concerns surrounding future career progression [39–40]. These additional stressors will have impacted foundation doctors experiences on the UKFP and as such, will likely have affected the results.

Ethical approval for this study was granted through the Research Ethics Committee (REC) based within the Health Research Authority in July 2023 (IRAS number: 328853).

### Setting & participants

All participants who took part in this study were foundation doctors within one NHS Foundation Trust. They worked at either one of two district general hospitals within the trust. These participants were either working as a Foundation Year 1 doctor (first year working as a doctor) or as a Foundation Year 2 doctor (second year working as a doctor). Each doctor would rotate to a different department every four months across various medical, surgical and primary care rotations.

### Recruitment

Foundation doctors were recruited via an email sent out through the Postgraduate Medical Education (PGME) department. The reason the PGME department were chosen to send out the emails was due to their frequent correspondence with the foundation doctors and their ability to contact all the foundation doctors within the trust via email. These emails included a participant information sheet and a participant consent form. The email also invited the foundation doctors to contact the researcher if they were interested in participating. Consent was obtained from all participants prior to their participation in the study.

**Table 1:** Inclusion and exclusion criteria

Inclusion Criteria	Exclusion Criteria
Doctors must be on the Foundation Programme during the 2023/2024 year.	Doctors must not have joined the programme later than August 2023.
Both doctors who had chosen to work full-time or part-time were included.	Doctors who were on the Foundation Programme outwith the trust were not eligible.

### Data collection

Once consent was obtained from the participants, a time was arranged to conduct the semi-structured interviews over Zoom. These interviews utilised an interview guide which was developed for this study (see supplementary document). Unlike the participant information sheet, the interview guide did contain questions that directly relate to burnout. The reason the participant information sheet did not mention burnout was to try and prevent participants having preconceived ideas about how their experiences on the UKFP and burnout related.

The interviews were conducted over Zoom as this provided flexibility and insured that interviews did not conflict with foundation doctors work commitments. Semi-structured interviews were chosen as it ensured that open ended questions could be asked and it enabled participants the time to explain their thoughts in detail [36]. Questions in the interviews focussed on participants experiences on the UKFP and their experiences of burnout whilst working as a newly qualified doctor. This meant the participants had the opportunity to discuss their lived experiences without being led by the interviewer or without being interrupted [41–42]. The semi-structured nature of the questions ensured that the interview remained relevant to the research question whilst it also afforded the participants the time to discuss issues which may have been sensitive or difficult to discuss [43]. As a result, the interviews provided a detailed insight into newly qualified doctors experiences on the UKFP and how these experiences related to their own experiences of burnout.

Each interview was transcribed after it took place. The interviews took place between October 2023 and April 2024. The length of time of the interviews ranged from between 11 min and 29 min. The average length of the interviews was 19 min and 51 s. The median length of the interviews was 19 min and 16 s.

### Data analysis

All interviews were manually transcribed by the researcher before being checked again to ensure accuracy. Transcriptions were then re-read by the researcher to ensure familiarity with the data. This data was then coded. NVivo 12 (QSR International) was used to support management and identify codes within the data. As the research aimed to address a specific research question only data which was relevant to this question was coded [34–35]. Once the data was coded, codes were then organised into themes based upon any overlapping patterns or meaning that was drawn from the codes. All themes were then refined and ensured that they related to the original research question [44, 45]. Once the themes were established, participants’ quotes were used to illustrate the themes within the results [34, 35, 46]. The purpose of this approach was to identify patterns within the data and use this to formulate an understanding of newly qualified doctors experiences on the UKFP and this programme related to their experiences of burnout.

Although the researcher ensured that they rigorously followed Braun and Clarke’s six-step approach to this analysis, only one researcher carried out the analysis. Therefore, it is important to note that the analysis is only coming from one perspective [35, 47]. However, a reflexive thematic analysis can be carried out by one researcher. This is because a reflexive thematic analysis draws upon the experience of the researcher to gain insight into the data [48–49].

### Reflexivity

As the researcher it is important to acknowledge that my own experiences and knowledge influenced the interpretation of the data. This is also relevant due to the constructivist paradigm of this research which ensures that it is the researcher’s responsibility to draw meaning from the data collected from the interviews [34]. Therefore, the data may be influenced by my own experiences as a foundation doctor in the trust. As a result, I am also aware of how burnout has impacted previous foundation doctor colleagues whilst on the UKFP.

## Results

**Table 2:** Participant characteristics

Participant Characteristics		
Number of Participants	7	
Gender	Male − 1	Female − 6
Medical School Entry Level	Undergraduate − 4	Postgraduate − 3
Foundation Year	Foundation Year 1–2	Foundation Year 2–5
Experienced Burnout	Yes − 7	No − 0
Full-time status on the UKFP	Yes − 4	No − 3

### Theme 1: understanding burnout

Participants were asked to define burnout in their own words in an attempt to understand each participants own meaning of the term burnout. All doctors interviewed had a similar definition that outlined the work component of burnout and how being overworked led to both physical and mental exhaustion.

“Burnout is complete exhaustion because of work whether that’s through employment or caring, it’s complete physical and mental exhaustion. This means you’re physically tired, you [get] out of healthy routines and don’t have time for hobbies. It’s just work, sleep, work, sleep, work, sleep rather than [doing] anything fulfilling.” **Doctor 2**

“I think it’s not working as well as you could. I think it’s being exhausted by the schedule and not having a work life balance. This leads to you not doing your work as well or as efficiently. You end up dreading going to work and looking forward to leaving because it’s just mentally exhausting.” **Doctor 5**

However, it was clear that some participants were drawing from their own lived experiences of burnout to form their definitions, they were not simply regurgitating a learned definition. This was clear because some participants would include how they managed their own burnout within their own definition.

“Burnout is mainly work related. When you get overwhelmed with stress and the job can lead you to be physically, mentally and emotionally drained to the point where you’re not functioning normally. And it usually leads people to getting to a crisis point and then having to take time off work to recuperate and reset and have support from their own GP and wellbeing services.” **Doctor 3**

Foundation doctors were then asked if burnout was something they were aware of whilst on the UKFP, including whether they themselves had experienced burnout. The majority of doctors were able to discuss their own experiences of burnout whilst working as a foundation doctor. Doctors often blamed the workload during the UKFP for directly for causing this phenomenon. When participants were asked to provide details of their own experiences of burnout, doctors linked their experiences of burnout with their negative experiences of working as part of the UKFP.

“I felt like I was getting overwhelmed with tasks and then I was having to do on-calls at the hospital. As soon as I left home in the morning I would just be like I have to 12 hours again and during the whole day I would just be overwhelmed.” **Doctor 1**

“Oh yeah, absolutely. I think I’m feeling [burnt out] now, to be honest. I don’t really look forward to going to work and I just prioritise my time outside of it. I think I’m doing the minimum amount I need to do [to get through the UKFP].” **Doctor 5**

### Theme 2: the effects of high stress rotations

As foundation doctors rotate into different specialities every 4 months, each doctor’s experience of the UKFP varies. However, there are certain rotations on the UKFP which are known to be more demanding and this can provide challenges for foundation doctors. In particular, medical rotations are seen to be one of the toughest components of the UKFP.

“After my experience in my medical rotation it’s kind of made me never want to work in a hospital medical department again. I didn’t like the working environment, I didn’t like the on-calls and sometimes you end up getting bombarded with [so many] bleeps you can’t get anything done.” **Doctor 3**

Some doctors already had negative views of these rotations before they had started working in the speciality. As a result, doctors were often fearful of starting these rotations and this in turn would heighten their anxieties prior to entering the department. Essentially these rotations were priming them to be at an increased risk of burnout.

“[My experience of medicine] was definitely like a baptism of fire. I went into medicine in a [district general hospital] with appalling staffing. With my first job, I knew it was going to be bad because I did my assistantship there, so the last part of my medical degree was on that ward so I knew what I was getting into but it was still a big shock to the system.” **Doctor 2**

As a result, medical rotations were typically considered to result in a more stressful day to day experience for foundation doctors. This occurred for a variety of reasons. The most common reasons given by foundation doctors were reduced staffing levels and a perceived lack of senior support on the wards. Despite these issues being known about they were never resolved and they were widely accepted within the department. This resulted in foundation doctors consistently working in a more stressful environment and an environment that had no hope of changing.

“Certain rotations obviously I found harder in terms of the sheer workload and maybe less support. I think I found my medical rotation quite hard in F1 because of the ward cover shifts overnight. I think that’s the hardest thing I found. The senior med regs tried to be supportive, but obviously their workload was so high as well, it was tough as an F1. The rotations I’ve done and what experiences I’ve had has showed me what kind of specialty I want to go into. It’s made me think I don’t want to do medicine or work in the hospital or anything like that.” **Doctor 6**

Foundation doctors also raised concerns that certain rotations had become associated with senior staff bullying junior doctors. This resulted in some foundation doctors going through the experience of being bullied on a daily basis on the wards. Often these senior staff were responsible for training these newly qualified doctors. Whilst these doctors faced the challenges of recurrent bullying they also lost out on training opportunities. Doctors who rotated through this speciality attribute their experience of being bullied as one of the key factors which caused substantial stress and their feelings of burnout. As a result, these doctors were put under a constant stress and it got to the point where the environment became impossible to work in.

“Me and another [foundation year 1 doctor] were the only doctors that had started on the ward on our induction day, and the consultant just left us to it and he would make us do the ward round on our own, despite us both raising concerns given that [we were only foundation year 1 doctors].[Once we raised concerns] it just escalated from there. I started having to make exception reports and get the guardian of safe working involved. And then he started to make our life a bit difficult on the ward. And that just affected my working life. I didn’t want to come to work. I’d be crying in the toilets because he’d be passive aggressive, he would threaten to not sign us off for our rotation because we’d raised concerns about him and [he] would make it a very awkward working environment. To the point where I didn’t want to come to work. I was crying to my educational supervisor. And I ended up having to get moved off of that rotation because of how I felt. And it spilled over into my personal life. I started getting heart palpitations. Because of the stress it all. It was a horrible experience.” **Doctor 3**

### Theme 3: work and personal life balance

Due to the rigid structure of the UKFP, many foundation doctors found the experience of managing their working lives as a newly qualified doctor alongside their personal lives particularly stressful. As a result of this stress, many doctors found the experience overwhelming.

Towards the end of my first foundation job there was like no staff. There was [one] point where I was doing a ward round for 27 patients on my own. And I went to [the Junior Doctor Forum] and I must’ve looked shocking because three separate people that had also been on the [Junior Doctor Forum] physically came to the ward [afterwards] to check that [I was alright] and they were like it’s fine, go sit down. And I was like, no, I just need to carry on. It’s fine. They were like no you need to stop. They brought me food and stuff, which was really nice. But I must have looked horrendous for them to actually come out from their office or their clinical work or wherever they were all coming from. And there was personal things as well. Like the day that I was on the ward on my own and my gran passed away. I just picked up a voicemail from my dad, like, yeah, it’s happened. And then I had to go back to seeing all the patients. And I was thinking this is horrendous  but there was literally no one else, so I couldn’t go home. **Doctor 2**

Some foundation doctors felt that they had no choice but to take time away from training. This time away from work would delay their training and highlighted that taking time was viewed by the training programme as an acceptable way to manage burnout.

“It was the balance between the difficult home life and the foundation program that led to me [having] quite a bit of time off. I’ve had about 14 weeks in total off. It’s difficult to really know if that is burnout in the technical sense, [but] the job has burnt me out [and] I would certainly say I experienced the symptoms of burnout.” **Doctor 7**

Foundation doctors noted that the day-to-day stressful experiences they had on the UKFP were bleeding into their personal lives. For some newly qualified doctors this impacted both their mental health and physical health. As a result, specific experiences on the UKFP resulted in them reaching a crisis point which required time off work.

“It would get to the point where I dreaded coming to work. I wasn’t sleeping well at night. I got physical symptoms such as the heart palpitations. I’d be crying at home every night, not wanting to come back to work the next day. In a way, I think I got a bit of PTSD and didn’t want to come back to the hospital.  [It got] to the point where I didn’t want to come to work at all. I was crying to my [Educational Supervisor] and I ended up having to get moved off that rotation because of how I felt essentially. And it spilled over into my personal life.” **Doctor 3**

### Theme 4: protective factors against burnout

Despite many newly qualified doctors finding the UKFP a stressful experience, participants did highlight aspects of the UKFP them to feel supported. One such aspect was that many foundation doctors highlighted that the UKFP provided a sense of community. Some foundation doctors found that this sense of community helped them to get through the UKFP more generally.

“I think the main thing that I think is a positive is the sense of community, like that kind of family feeling about being around other F1s. I think the fact that you’re all going through the same thing and it’s a very intense thing to be going through, I think that really makes people stick together. I [have] found that the other F1s are all just really lovely and are always there for each other to be like an ear to listen to. You can discuss complaints and positive things from personal life and work life.” **Doctor 7**

Some doctors who were experiencing high levels of stress on the UKFP tried to adapt the programme to reduce their levels of burnout. One option which was available to them was to reduce the number of hours they worked as part of the UKFP. This would mean that they would be on the UKFP for a longer period of time but they would work less hours each week. Participants who did this found that this helped to protect them from further experiences of burnout.

“I’ve heard many colleagues mentioning how stressed they are and that’s a really common statement from ‘full-timers.’ I was less than full time and this is the thing that saved me from [burnout].” **Doctor 4**

“When I’ve come back to work, after my time off, I’ve gone less than full-time. I think that makes me less worried about burnout because I’m hoping to stay less than full-time to protect myself from that. So yes, I guess that is an ongoing concern in the sense if I went back to full-time, I’m not sure I would cope.” **Doctor 7**

## Discussion

Burnout is widely recognised to be increasing amongst doctors who are at the beginning of their careers [50]. This aligns with foundation doctors in the UK who continue to report higher levels of burnout [51]. However, there is limited research which addresses why foundation doctors are at a particular risk of burnout. Participants in this study were able to address this. They were able to highlight some of the broader issues in the NHS which they believed were leading to their own experiences of burnout, as well as some issues which were specific to the UKFP. Participants also highlighted aspects of the UKFP which they found reduced their experience of burnout.

It could be argued that because newly qualified doctors are facing an ever-increasing intensity of demands in their role this is resulting in them being put under more stress. Results from this research are in keeping with the Job-Demands Resource (JD-R) theory. This theory is often applied to try and explain causes of burnout within a workforce [52–53]. The JD-R theory suggests that if a work environment has a combination of high job demands and low job resources then this will inevitably lead to the staff who are working in this environment burning out [53]. This can be applied to newly qualified doctors working within the NHS. Newly qualified doctors now face ever increasing job demands and this leads to an increase in stress on the doctors. Then as there is no intervention at this point (e.g. more resources/staff becoming available to help), doctors develop maladaptive coping mechanisms to deal with working in this high stress environment. For example, doctors may develop a cynicism to their work environment. It is these maladaptive coping mechanisms which are often considered as the first symptoms of burnout [53–54].

Newly qualified doctors already had concerns about burnout prior to starting their first jobs in the NHS [33, 55]. This is likely due an increased awareness of the issue [55]. Participants in this study were able to discuss their own understanding of the term burnout. Participants were also able to describe their own experiences of burnout and how this related to their experiences on the UKFP. This is not surprising given previous research has highlighted burnout is increasing amongst doctors, especially those at the beginning of their career [5, 6]. Concerningly, participants directly attributed their experiences of burnout to the UKFP and more broadly, to working in the NHS.

One potential cause of burnout in foundation doctors is the rotational nature of the UKFP. Rotations have led to doctors having vastly different day-to-day experiences during their training. For example, many participants perceived some specialities, medical specialities in particular, to be more demanding than others which led to participants feeling that they were at a greater risk of burnout. This is perhaps unsurprising as previous research found that one of the biggest causes of emotional exhaustion and distress for foundation doctors was excessive workload. Participants reported that work demands during medical rotations were often exacerbated by reduced staffing levels and a lack of senior support [18]. This is in keeping with previous research which highlights that a dysfunctional work environment can double the rate at which trainee doctors report experiencing burnout [56]. This example also highlights that the rotational element of the UKFP could be contributing to early career burnout, especially for those doctors who have been allocated to a higher number of rotations which are typically considered to be more demanding.

Rotations may also cause anxiety if foundation doctors rotate into a work environment where colleagues have previously raised concerns [55, 57]. Participants noted one rotation in particular where there were concerns of senior staff bullying doctors. Participants directly linked their experiences of being bullied by senior staff on the UKFP with their experiences of burnout. This is perhaps unsurprising given that burnout is directly linked to prolonged exposure to emotional stress [55]. Worryingly, these experiences are not uncommon, as 20% of doctors in training report being bullied by colleagues within the NHS. Whilst bullying is not unique to the Foundation Programme, it remains a significant issue for doctors in training in the NHS and has proven links to the burnout [58].

The UKFP is an unflexible training programme. Doctors are allocated to a hospital, change job roles every four months and told to work on rotas where they have little say about the shifts they work [59]. Due to this rigidity, the UKFP can make it challenging for doctors to successfully navigate their personal lives alongside their work lives. For example, foundation doctors who have caring responsibilities may find it particularly challenging to find childcare when they are on-call [60]. If doctors are having difficulties in their home life this will often have a knock-on effect and result in the doctor struggling at work. These competing priorities can cause doctors additional stress, often leading to burnout. As a result, some participants felt that they had little choice but to take time away from training.

As all of the participants were aware that newly qualified doctors are at risk of burnout, many identified different approaches to reduce their risk. One reported method was to reduce their working hours to less than full-time. All three participants who had reduced their hours stated that they would be reluctant to go back to full-time at any point in their medical training. They noted that returning to full-time work would likely result in them experiencing burnout. This is in-keeping with previous research. Jung et al. (2023) noted that physicians who are experiencing burnout often attempt to mitigate against it through planning a reduction in work hours [61]. Doctors who do reduce their work hours often report that this as an effective way to reduce their risk of burnout [62]. This was also noted in the ‘F3 Phenomenon Report’ were the introduction of the option for foundation doctors to work less than full-time was considered to have “the potential to prevent or reduce burnout experienced through foundation training” [33].

Participants acknowledged that being part of the UKFP provided a sense of community. Foundation doctors noted experiences where they took the time to discuss the challenges of the role with colleagues helped to reduce their stress. Previous research has highlighted that the emotional support from other colleagues can help foundation doctors manage the day-to-day challenges of the role. It was also considered protective against declines in mental health [18]. This is likely because feelings of belonging and collegiality have been shown to promote wellbeing within a group. Promoting wellbeing is widely recognised as a way to counteract stress [63–64]. These findings highlight the importance of bringing foundation doctors together, for example through weekly teaching sessions. This provides foundation doctors the opportunity to seek support from each other and work through any challenges they are having.

### Limitations

A limited number of foundation doctors took part in this study. However, phenomenological research can have a smaller number of participants provided the data gathered provides an understanding of a particular phenomenon. One mechanism for achieving this level of depth in each interview is to ensure open ended questions are asked throughout [65–66]. This allows participants the opportunity to discuss their lived experiences without being led by the interviewer or without being interrupted [41–42]. This approach was utilised during this study.

The number of participants needed within a qualitative research project can be challenging to identify [62]. One approach to identifying the number needed is to establish when a saturation point is being achieved. Whilst this process is subjective, it can be done through recognising when no new data is being gathered through the interviews [67]. Within this study, the saturation point became apparent after 7 interviews. This is also in keeping with Braun and Clarke’s thematic analysis guidelines which also suggests recruiting between 6 and 10 participants for interviews for a small research project [48]. Previous research has also suggested that themes within a thematic analysis become apparent after interviewing 6 participants [68].

However, as establishing a saturation point can be subjective, the reliability of this research could have been increased if there were more researchers involved to discuss this with. Other researchers would have also helped with the data analysis and the establishment of themes. In attempt to overcome this, this research was frequently presented at the Research Assurance Group within the NHS trust. The Research Assurance Group provided feedback to the researcher and helped to identify any issues with the study.

Whilst this research only took place at two hospitals within one NHS trust, participants from this study were part of the wider UKFP. Therefore, their experiences are likely to overlap with other newly qualified doctors within the UK. However, this research may have reduced transferability to an international audience as the UKFP and the NHS are unique to the UK. Although it is important to consider burnout in newly qualified doctors remains an issue in many countries [1].

### Further research

Further research is needed to identify effective support measures which can be implemented within the UKFP and used to support doctors as they begin their career in the NHS. Examples of this research would include studies which focus on establishing the benefits of peer-to-peer support on the UKFP as well as research which explores how the UKFP could change as a result of the increasing demands being placed upon the NHS.

## Conclusion

Burnout continues to remain a significant concern amongst newly qualified doctors. Most doctors in this study could describe a detailed account of their own experiences of burnout. Some participants believed the UKFP directly contributed to their burnout through factors such as rotational training. However, there were also some specific aspects of the UKFP which were thought to be protective against burnout.

Burnout was also attributed to issues which are prevalent throughout the NHS such as bullying and high work demands. This may explain why burnout is on the rise in this group. These newly qualified doctors are starting their first role in what has become an increasingly strained NHS and unfortunately as the work demands continue to increase so does the risk of burnout in newly qualified doctors.

## Electronic supplementary material

Below is the link to the electronic supplementary material.

Supplementary Material 1

Supplementary Material 2

Supplementary Material 3

Supplementary Material 4

Supplementary Material 5

## Data Availability

The datasets generated and analysed during the current study are not publicly available due privacy or ethical restrictions but are available from the corresponding author on reasonable request.

## Abbreviations

UKFP: United Kingdom Foundation Programme
NHS: National Health Service
WHO: World Health Organization
GMC: General Medical Council
BMA: British Medical Association
PGME: Postgraduate Medical Education
