# Supplementary material for: A thematic analysis of newly qualified doctors’ experiences of burnout
Source: BMC Med Educ. 2025 Apr 7;25:494. doi: 10.1186/s12909-025-07076-z (PMC11978160; doi:10.1186/s12909-025-07076-z)
Supplement: Supplementary file 1 — Supplementary Material 1 [file 12909_2025_7076_MOESM1_ESM.docx]

13^th^ October 2024

BMC Medical Education

I would be grateful if you would consider my article: ‘A Thematic Analysis of Newly Qualified Doctors Experiences of Burnout’ for publication in BMC Medical Education.

In this qualitative research we explored newly qualified doctors experiences of burnout as they begin their postgraduate training whilst working within the NHS. Whilst there is already detailed literature outlining that burnout is on the rise amongst doctors, there is limited research which explores why newly qualified doctors appear to be at a particular risk of burnout. This research has outlined aspects of working within the NHS which could be considered as contributory factors to burnout as well as aspects of the UK Foundation Programme itself which have increased the risk. Participants were also able to identify aspects of their training which were considered protective against burnout such as the community aspect of working with other foundation doctors and the option to reduce their working hours to less than full-time.

This research is of interest to clinicians responsible for the education of newly qualified doctors and newly qualified doctors themselves. It is also of interest to the UKFPO who are responsible for the oversight and development of the UK Foundation Programme.

As the author I have read and approved the final manuscript and have no direct or indirect commercial or financial incentive associated with publishing the article. There are no conflicts of interest. This work has not previously been published and is not being considered to be published elsewhere.

Your sincerely,

Dr Colin Kilday

Resident Doctor

University of Hull, Hull, UK
