# Supplementary material for: A thematic analysis of newly qualified doctors’ experiences of burnout
Source: BMC Med Educ. 2025 Apr 7;25:494. doi: 10.1186/s12909-025-07076-z (PMC11978160; doi:10.1186/s12909-025-07076-z)
Supplement: Supplementary file 2 — Supplementary Material 2 [file 12909_2025_7076_MOESM2_ESM.docx]

**Participant Information Sheet**

**An investigation into the experiences of foundation doctors on the Foundation Programme**

This is an invitation for you to take part in a research study. Please read this sheet. You are welcome to ask questions by emailing the researcher (contact details can be found below).

In this research study we will use information provided by yourself. We will only use information that we need for the research study. We will let very few people know your name or contact details, and only if they really need it for this study.

Everyone involved in this study will keep your data safe and secure. We will also follow all privacy rules.

At the end of the study, we will save some of the data as a transcription and quotations from the interviews which will be used as part of the results of the study.

We will make sure no-one can work out who you are from the reports we write.

You may withdraw consent and discontinue your participation in the study at any time without prejudice, your data will then be deleted.

**Contact Details of Researcher**

Dr Colin Kilday

[colin.kilday@nhs.net](mailto:colin.kilday@nhs.net)

**The purpose of the study**

To investigate the experinces of foundation doctors during the foundation programme.

**Your role in the study**

You were chosen to take part in the study because you were part of the Foundation Programme during the 2023/2024 year.

**Participation in Study**

To take part in the study you should reply to the request for participants issued by the PGME department. You will also need to sign the consent form which has been issued with this participation information sheet if you would like to take part.

**Do I have to take part in the study?**

No, you have a choice whether you want to participate in the interview. You will be asked if you wish to take part in the interview. Should you agree and consent to take part in the interview you will contacted via email to arrange the interview. If you do decide to take part, you will be given this information sheet to keep. Should you have any questions these can be asked via email or prior to the beginning of the interview.

*Please note that you can withdraw your consent at any time, and it can be withdrawn without giving a reason.*

**What are the possible risks of taking part?**

This study is highly unlikely to expose you to any known potential risks or discomforts. However, should you have any concerns please contact the researcher or the PGME department.

**What will happen to me if I take part?**

You will be asked whether you wish to take part in an interview which would take place over Zoom. If you agree to take part in the interview, this will focus on questions around burnout, the Foundation Training Programme and your future career aspirations.

The interview would be recorded. The information discussed would be anonymised and each interviewee will be assigned a number and the data will not be recorded with the participant’s name. The time arranged for the interview would depend upon the availability of both you as the participant and myself as the interviewer.

The interview recordings will be stored on an NHS drive and only accessed through NHS laptops and computers. Only myself, as the researcher, and the Research & Development Department would have access to these interviews. Unfortunately, it would not be possible to take part in the study without the interview being recorded. This is because the interviews will need to be transcribed. All interviews would be transcribed by myself, the researcher. No identifying information would be noted on the transcribed document. The information would be transcribed within a 2-week period from the date of interview.

Although unlikely, if something is said that could raise a potential concern about fitness to practice and/or safeguarding, specific details will be gathered and shared with PGME and the Foundation Year Training Programme Director. They would then navigate subsequent escalation to the relevant individuals and committees.

**What are your choices about how your information is used?**

You can stop being part of the study at any time, without giving a reason, but we will keep information about you that we already have. We need to manage your records in specific ways for the research to be reliable. This means that we won’t be able to let you see or change the data we hold about you.

**Where can you find out more about how your information is used?**

You can find out more about how we use your information:

o at [www.hra.nhs.uk/information-about-patients/](http://www.hra.nhs.uk/information-about-patients/)

o by asking one of the research team.

o by sending an email to *colin.kilday@nhs.net*

**What happens when the research study stops?**

There are no implications for you; if the research has raised areas which you wish to discuss further you should contact either the researcher or the PGME department.

**What if something goes wrong?**

If anything occurs, you should seek the advice of the researcher, PGME or the Foundation Year Training Programme Director.

**Will my taking part in this study be confidential?**

We will need to use information from yourself for this research project.

This information will include:

- Age Range
- Gender
- Years of University Education
- Plans after completing Foundation Training Programme (e.g., Speciality Training, Locum, Clinical Fellow, etc.)

People will use this information to do the research or to check your records to make sure that the research is being done properly.

People who do not need to know who you are will not be able to see your name or contact details. Your data will be anonymised and you as the participant will be referred to by your assigned number (e.g., Dr 1) on documentation/results.

We will keep all information about you safe and secure.

Once we have finished the study, we will keep some of the data so we can check the results. We will write our reports in a way that no-one can work out that you took part in the study.

Should this research be published, direct quotations from participants would be used within the publication. Any quotes used would be anonymised and participants would be referred to by their assigned numbers.

**Who is organising and funding the research?**

The study is being organised by Dr Colin Kilday.
