# Supplementary material for: A thematic analysis of newly qualified doctors’ experiences of burnout
Source: BMC Med Educ. 2025 Apr 7;25:494. doi: 10.1186/s12909-025-07076-z (PMC11978160; doi:10.1186/s12909-025-07076-z)
Supplement: Supplementary file 5 — Supplementary Material 5 [file 12909_2025_7076_MOESM5_ESM.docx]

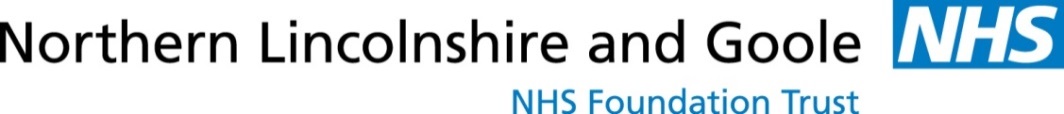


**Semi-Structured Interview Questions**

**Foundation Programme**

- How have you found your experience as a Foundation Doctor?
- Can you tell me about some positive experiences on your Foundation Training Programme?
- Can you tell me about some negative experiences on your Foundation Training Programme?
- Is there anything you would change about your experiences of the Foundation Training Programme if you had the opportunity?
- Have your views on Foundation Training changed since you were in Medical School?
- If yes, in what ways?

**Burnout Definition**

- Can you describe the term burnout?

**Identifying Burnout**

- What do you think are the signs and symptoms of burnout in a Foundation Doctor?
- Do you feel burnt out because of your work?
- Do you think you could tell if you were experiencing burnout?
- Do you think you would pick up if one of your Foundation Doctor colleagues was experiencing burnout?
- What is the difference between burnout and being burnt out?

**Burnout Concerns**

- Do you have any concerns about burnout?
- Are you concerned about future training programmes leading to burnout?
- Do you feel like taking a break from training would prevent burnout?

**Support**

- During your foundation training have you experienced burnout?
- If yes, have you discussed this with anyone?
- Were you offered support?
- If you were ever to experience burnout out would you know who to speak to?
- Would you have any concerns about discussing burnout whilst on the Foundation Programme?

**Progression**

- Do you feel like the Foundation Training Programme itself has contributed to burnout?
- Were you concerned about burnout during medical school?
- Do you feel medical school contributes to burnout of doctors?
- Are you worried about burnout as you progress through your career as a doctor?
- Do you think there are any ways to prevent burnout?

**Career in Medicine**

- What are your plans after you finish the foundation training programme?
  - Has burnout contributed to these plans?
  - Has anything else contributed to your plans?
- Has your experience as a Foundation Doctor impacted your thoughts on a career in medicine?
  - If it has, in what ways?
- Has burnout impacted your views on a career in Medicine?
  - If it has, in what ways?

**Conclusion**

- Do you have any questions or do you have any comments that you would like to add to this interview?
