# Supplementary material for: A thematic analysis of newly qualified doctors’ experiences of burnout
Source: BMC Med Educ. 2025 Apr 7;25:494. doi: 10.1186/s12909-025-07076-z (PMC11978160; doi:10.1186/s12909-025-07076-z)
Supplement: Supplementary file 4 — Supplementary Material 4 [file 12909_2025_7076_MOESM4_ESM.pdf]

Ymchwil Iechyd a Gofal Cymru
Health and Care Research Wales

NHS
Health Research Authority

Dr Colin Kilday
Orthopaedic SHO (Junior Doctor)
Northern Lincolnshire & Goole Foundation Trust
Goole & District Hospital
Woodland Avenue
Goole
DN14 6RX

Email: approvals@hra.nhs.uk
HCRW.approvals@wales.nhs.uk

23 June 2023

Dear Dr Kilday

HRA and Health and Care Research Wales (HCRW) Approval Letter

**Study title:**	**An investigation into the impacts of burnout on Foundation Doctors**
**IRAS project ID:**	**328853**
**Protocol number:**	**N/A**
**REC reference:**	**23/PR/0627**
**Sponsor**	**Northern Lincolnshire & Goole Foundation Trust**

I am pleased to confirm that **HRA and Health and Care Research Wales (HCRW) Approval** has been given for the above referenced study, on the basis described in the application form, protocol, supporting documentation and any clarifications received. You should not expect to receive anything further relating to this application.

Please now work with participating NHS organisations to confirm capacity and capability, in line with the instructions provided in the "Information to support study set up" section towards the end of this letter.

How should I work with participating NHS/HSC organisations in Northern Ireland and Scotland?

HRA and HCRW Approval does not apply to NHS/HSC organisations within Northern Ireland and Scotland.

If you indicated in your IRAS form that you do have participating organisations in either of these devolved administrations, the final document set and the study wide governance report (including this letter) have been sent to the coordinating centre of each participating nation. The relevant national coordinating function/s will contact you as appropriate.

Please see IRAS Help for information on working with NHS/HSC organisations in Northern Ireland and Scotland.

How should I work with participating non-NHS organisations?
HRA and HCRW Approval does not apply to non-NHS organisations. You should work with your non-NHS organisations to obtain local agreement in accordance with their procedures.

What are my notification responsibilities during the study?

The *"After HRA Approval – guidance for sponsors and investigators"* document on the HRA website gives detailed guidance on reporting expectations for studies with HRA and HCRW Approval, including:

- Registration of Research
- Notifying amendments
- Notifying the end of the study

The HRA website also provides guidance on these topics and is updated in the light of changes in reporting expectations or procedures.

Who should I contact for further information?
Please do not hesitate to contact me for assistance with this application. My contact details are below.

Your IRAS project ID is **328853**. Please quote this on all correspondence.

Yours sincerely,
Claudia Bywater

Approvals Manager

Email: approvals@hra.nhs.uk

Copy to: *Rachel Pollard, Northern Lincolnshire & Goole Foundation Trust*

List of Documents

The final document set assessed and approved by HRA and HCRW Approval is listed below.

Document	*Version*	*Date*
Interview schedules or topic guides for participants [Interview Question Guide (An investigation into the impacts of burnout on Foundation Doctors)]	1	05 June 2023
IRAS Application Form [IRAS_Form_26052023]		26 May 2023
Participant consent form [Participant Consent Form]	2	05 June 2023
Participant information sheet (PIS) [Participant Information Sheet (Version 3)]	Version 3	22 June 2023
Research protocol or project proposal [Research Protocol (An investigation into the impacts of burnout on Foundation Doctors)]	2	05 June 2023
Summary CV for Chief Investigator (CI) [Research CV - Dr Colin Kilday]		18 April 2023

IRAS project ID	328853

Information to support study set up

The below provides all parties with information to support the arranging and confirming of capacity and capability with participating NHS organisations in England and Wales. This is intended to be an accurate reflection of the study at the time of issue of this letter.

Types of participating NHS organisation	Expectations related to confirmation of capacity and capability	Agreement to be used	Funding arrangements	Oversight expectations	HR Good Practice Resource Pack expectations
This is a single site study sponsored by the participating NHS organisation therefore there is only one site type.	The single participating NHS organisation of this type is also sponsoring the research. You should work with your sponsor R&D office to make arrangements to set up the study. The sponsor R&D office will confirm to you when the study can start following issue of HRA and HCRW Approval.	This is a single site study sponsored by the participating NHS organisation therefore no agreements are expected.	No external study funding has been sought	Principal Investigators are expected to be in place at participating NHS / HSC organisations where locally employed staff take responsibility for research procedures.	Where an external individual is conducting only research activities that are limited to access to staff, or staff data (in either identifiable or anonymised form), or anonymised patient data then a Letter of Access is required only if these activities will take place in NHS facilities. This should be issued be on the basis of a Research Passport (if university employed) or an NHS to NHS confirmation of pre-engagement checks letter (if NHS employed). Where these activities will not take place in NHS facilities then no arrangements under the HR Good Practice Pack are required.

Other information to aid study set-up and delivery

This details any other information that may be helpful to sponsors and participating NHS organisations in England and Wales in study set-up.
The applicant has indicated that they intend to apply for inclusion on the NIHR CRN Portfolio**.**
